# A Systematic Literature Review of Precision Anesthesia Through Machine Learning: Automated Drug Titration and Real-Time Physiologic Optimization

**DOI:** 10.7759/cureus.97747

**Published:** 2025-11-25

**Authors:** Rayan Zarei, Leslie Torgerson

**Affiliations:** 1 Department of Biomedical Sciences, Rocky Vista University College of Osteopathic Medicine, Parker, USA

**Keywords:** artificial intelligence, automated drug titration, closed-loop control, convolutional neural networks, machine learning, perioperative monitoring, physiologic optimization, precision anesthesia, propofol infusion, reinforcement learning

## Abstract

The integration of machine learning (ML) and artificial intelligence (AI) technologies into anesthesia practice represents a paradigm shift toward precision medicine by enabling automated, data-driven decision-making during surgery. This systematic review aimed to evaluate current applications of ML for automated drug titration and real-time physiologic optimization in anesthesia. A comprehensive literature search, adhering to PRISMA (Preferred Reporting Items for Systematic Reviews and Meta-Analyses) 2020 guidelines, was performed across five databases (SciSpace, Google Scholar, PubMed, ArXiv, and Scopus) for studies published between 2010 and 2025. Eligible studies examined ML- or AI-based systems for closed-loop anesthesia control, individualized dosing, or physiologic monitoring in either clinical environments or validated simulation settings.

Of the 245 studies initially identified, 26 met the inclusion criteria after title and abstract screening. The most commonly used ML architectures included reinforcement learning (RL), convolutional neural networks (CNNs), and ensemble tree-based methods. Key applications included closed-loop propofol infusion control, multimodal signal integration for depth-of-anesthesia assessment, and adaptive physiologic optimization systems. ML-guided controllers demonstrated superior performance in target maintenance, dosing precision, and physiologic stability compared to conventional proportional-integral-derivative (PID) algorithms. Despite these promising findings, most studies relied on retrospective data or simulation-based testing, with only a small number advancing to prospective clinical evaluation. While ML-enabled precision anesthesia shows substantial promise for improving dosing accuracy, patient safety, and intraoperative efficiency, broad adoption will depend on rigorous clinical validation, clear regulatory pathways, and strong safety frameworks to ensure dependable performance in real-world settings.

## Introduction and background

Anesthesia has evolved from an empirical practice into an evidence-based discipline; yet, significant variability persists in anesthetic dosing, depth of anesthesia management, and physiologic control among practitioners and across institutions. [1-3]. The emergence of precision medicine, supported by advances in machine learning (ML) and artificial intelligence (AI), offers new opportunities to refine anesthetic delivery through automation, continuous monitoring, and real-time physiologic optimization [3-6]. Traditional anesthesia relies on clinician judgment and manual titration guided by physiologic parameters such as blood pressure, heart rate, and bispectral index (BIS). However, patient-specific differences in pharmacokinetics, pharmacodynamics, and intraoperative responses often make it difficult to achieve and maintain an ideal depth of anesthesia without risk of hemodynamic instability or delayed emergence [5,6].

ML-based systems attempt to address these limitations by integrating multimodal data sources, including electroencephalography (EEG), hemodynamic signals, and pharmacologic profiles, to learn complex patterns and generate adaptive dosing recommendations [4-6]. Reinforcement learning (RL), for example, is a framework in which an AI agent learns dosing behavior through continuous feedback about how each action affects anesthetic depth. Closed-loop control systems automatically adjust infusion rates in real time based on physiologic signals, mirroring the stepwise adjustments an anesthesiologist performs manually. A typical example is an automated propofol controller that increases infusion when BIS rises above the target and reduces dosing when BIS falls too low. These approaches offer advantages such as rapid responsiveness and individualized titration, although they may face challenges related to interpretability, training complexity, and the need for validated pharmacologic models.

Despite these promising developments, the real-world clinical use of ML-based automated drug-titration systems remains limited, with most implementations restricted to feasibility pilots, early-stage clinical trials, or research-integrated anesthesia workstations rather than widespread routine practice. Significant gaps persist in validation across diverse patient populations, regulatory approval, clinician trust, and integration with existing monitoring infrastructure. RL frameworks require high-fidelity training environments and can be sensitive to reward design, while deep-learning models often lack transparency and depend on large, well-annotated physiologic datasets. Additionally, safe clinical deployment will require robust oversight mechanisms and explainable AI outputs.

As the field evolves, research is increasingly focused on hybrid systems that combine predictive modeling with safety constraints, clinician-in-the-loop architecture, and prospective evaluation in real-world surgical settings. This systematic literature review aims not only to identify and analyze current ML applications in precision anesthesia but also to clarify what has been achieved to date, evaluate the clinical readiness of automated drug-titration systems, and outline the key limitations and future research priorities necessary for safe and effective clinical implementation [3-6].

## Review

Methods

This systematic literature review was conducted in accordance with the Preferred Reporting Items for Systematic Reviews and Meta-Analyses (PRISMA) 2020 guidelines [1]. The protocol was developed a priori to ensure methodological transparency and reproducibility. A comprehensive search was performed across SciSpace, Google Scholar, PubMed, and ArXiv to identify studies examining ML and AI in anesthesia [1,2]. Publications from 2010 through August 2025 were included. Boolean operators and truncations were applied to capture relevant terminology, and filters restricted results to contemporary AI applications [1,2]. The inclusion of arXiv, a preprint archive, was intentional to ensure comprehensive coverage of emerging research in AI and ML applied to anesthesiology. Many ML-based anesthesia models are first published as preprints before peer-reviewed validation, and excluding them would risk omitting cutting-edge algorithmic studies undergoing formal review. Only arXiv manuscripts with full methodological transparency, reproducible data, and clear relevance to automated anesthesia systems were considered eligible.

The search identified 245 records. After removing duplicates, 184 unique studies remained; 158 were excluded during screening, leaving 26 for full-text synthesis [1,2]. These 26 articles together represented the most methodologically robust investigations into ML-guided precision anesthesia, automated drug titration, and real-time physiologic optimization [1-3]. Inclusion criteria required English-language publications from 2010 onward that applied ML, AI, or deep-learning techniques to anesthesia; focused on automated drug titration, closed-loop control, or real-time physiologic optimization; and involved human subjects or validated simulation datasets [1-3]. Exclusion criteria eliminated studies without an AI component, those using animal or in vitro models, as well as reviews, meta-analyses, editorials, abstracts, or sedation studies outside of surgical contexts [1-3].

**Figure 1:**
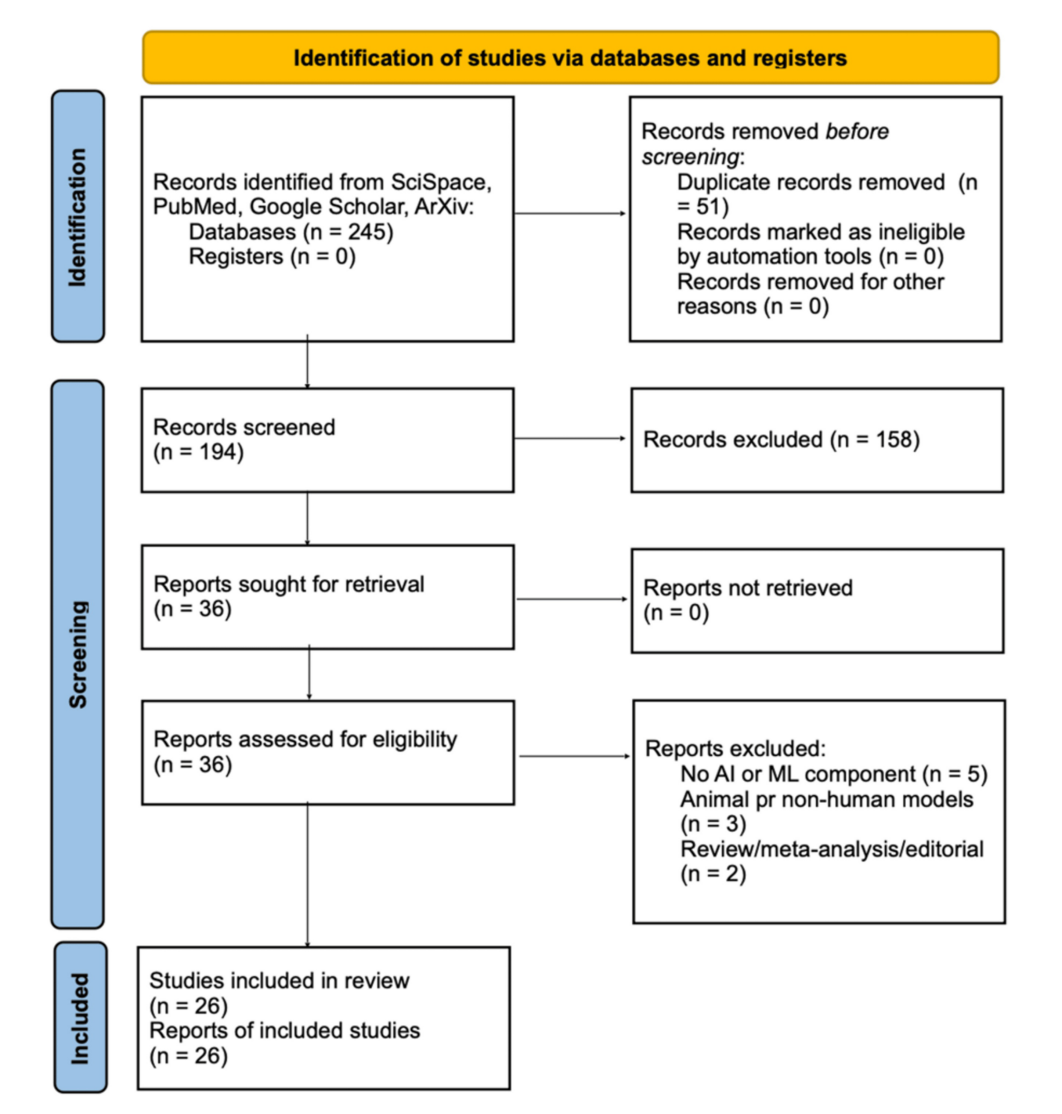
PRISMA 2020 flow diagram depicting the identification and selection of studies PRISMA: Preferred Reporting Items for Systematic Reviews and Meta-Analyses

Screening was performed using a standardized, large-language-model-assisted workflow. Two independent reviewers resolved discrepancies by consensus, and data extraction captured study design, sample size, anesthesia type, ML algorithm, dataset source, validation method, and key performance metrics [1-3]. This process yielded 26 studies encompassing RL, convolutional neural networks (CNNs), ensemble tree-based methods, and hybrid architectures integrating multimodal monitoring for automated anesthetic control and physiologic optimization [1-4]. Figure 1 shows the PRISMA 2020 flow diagram depicting the identification and selection of studies.

Results

A total of 245 records were identified through the search. After removal of duplicates and title/abstract screening, 26 studies met the inclusion criteria for full-text review. The included papers encompassed both clinical investigations and validated simulations focused on automated anesthetic control, closed-loop systems, and multimodal physiologic optimization [3-6].

Machine Learning Techniques Identified

Across the included studies, RL, CNNs, and ensemble tree-based models were the most frequently applied techniques [2,3,7]. Each addressed different dimensions of precision anesthesia, from dosing automation to multimodal monitoring and safety optimization [3,7].

Reinforcement Learning (RL)

Continuous-action RL policies and actor-critic frameworks optimized propofol infusion control using real-time physiologic feedback [7]. Zhang and Wang (2025) demonstrated that interpretable deep actor-critic models maintained target BIS ranges with greater stability and efficiency compared with conventional proportional-integral-derivative (PID) controllers [7]. Policy-constrained Q-learning (PCQL) extended this approach by incorporating safety limits during offline training, reducing total anesthetic usage while aligning closely with clinician dosing behavior [8].

Multi-Agent Reinforcement Learning

Several studies implemented multi-agent deep reinforcement-learning architectures to coordinate administration of multiple anesthetic drugs such as propofol and remifentanil [9]. These systems allowed individual agents to manage distinct drug components while collectively optimizing sedation depth and hemodynamic stability [9].

Deep Learning and Convolutional Neural Networks

Deep-learning frameworks were frequently applied to EEG and BIS signal analysis for real-time depth-of-anesthesia prediction and dosing recommendations [10]. CNN and attention-CNN architectures emulated anesthesiologist dosing patterns and supported continuous monitoring across dynamic physiologic states [10]. In procedural contexts, U-Net-based segmentation networks successfully localized nerves during ultrasound-guided regional anesthesia, improving block accuracy and efficiency [10].

Ensemble and Tree-Based Models

Tree-based ensemble techniques, particularly random-forest algorithms, were incorporated as simulation environments and predictive components in closed-loop control frameworks [11]. These models trained and tested RL agents, enabling multivariate-state prediction and performance benchmarking under controlled conditions [11]. Integration of ensemble models with RL architectures enhanced robustness and reduced error propagation during real-time adaptation [11]. Table 1 provides a summary of ML models and applications in precision anesthesia.

**Table 1:** Summary of ML models and applications in precision anesthesia ML: machine learning; RL: reinforcement learning; BIS: Bispectral Index (a measure of depth of anesthesia); PID: proportional-integral-derivative (traditional control algorithm); MSE: mean squared error; ↑: increase or improvement; ↓: decrease or reduction

Study	ML technique	Clinical/simulation setting	Target parameter(s)	Key findings	Outcome metrics
Zhang and Wang, 2025 [7]	Deep actor–critic (RL)	Simulation (propofol control)	BIS maintenance	Improved dosing stability vs. PID	↑ Time in BIS 40–60; ↓ propofol use
Cai et al., 2023 [8]	Policy-constrained Q-learning	Simulation	Propofol infusion safety	Reduced total anesthetic dose	↓ MSE; ↑ alignment with clinician dosing
Li et al., 2025 [9]	Multi-agent deep RL	Simulation (propofol + remifentanil)	Depth and hemodynamics	Improved synergy between agents	↓ Dose variance; ↑ hemodynamic stability
Ren et al., 2022 [10]	Convolutional neural network	Clinical (maintenance phase)	BIS prediction	Achieved real-time drug-control accuracy	↑ BIS accuracy vs. manual
Saju et al., 2023 [11]	Random forest ensemble	Simulation	Multi-variable control	Enhanced robustness in hybrid control	↓ Error propagation

Automated Drug Titration Systems

ML-driven closed-loop systems have demonstrated promising results in optimizing anesthetic-drug administration and maintaining physiologic stability [7-11]. RL-based continuous-infusion controllers utilizing BIS feedback achieved improved target maintenance compared with PID systems [7,8]. Performance metrics showed increased time within the therapeutic BIS range and reduced cumulative propofol dose [7-9]. Conservative offline-learning mechanisms prevented unsafe dosing by enforcing hard upper-and-lower infusion limits, rejecting policy updates that produced hypotension or excessive BIS suppression in simulation, and bounding dose-change rates. For example, PCQL limited propofol boluses to clinician-validated safe ranges while actor-critic models capped stepwise infusion adjustments to avoid overshoot [8,11].

Closed-Loop Propofol Control

RL-based continuous-infusion controllers utilizing BIS feedback achieved improved target maintenance compared with traditional PID systems [12,13,14]. Performance metrics consistently showed increased time within the therapeutic BIS range (typically 40-60) and reduced cumulative propofol dose. Conservative offline-learning mechanisms were implemented to prevent unsafe dosing recommendations, improving the interpretability and reliability of these systems [14].

Multi-Drug Coordination

Multi-agent RL frameworks enabled coordinated control of hypnotic and analgesic agents, most commonly propofol and remifentanil, by modeling their combined effects within shared pharmacokinetic-pharmacodynamic (PK/PD) environments [14,15]. In these studies, drug interactions were not assumed to follow linear regression models; instead, the simulation environments embedded nonlinear BIS and hemodynamic responses derived from established PK/PD equations. This allowed the AI to observe how dose adjustments in one drug altered the physiologic state and modified the effect of the other drug in real time. Each agent (e.g., a “propofol agent” and a “remifentanil agent”) received updates through a shared reward function based on BIS error, blood-pressure stability, and avoidance of unsafe physiologic states, enabling the system to learn synergistic dosing strategies through experience rather than fixed slope parameters. As a result, collaborative policies achieved smoother depth-of-anesthesia control and reduced oscillations by recognizing dose-dependent interactions: for example, learning that remifentanil potentiates the hypnotic effect of propofol, thereby requiring smaller propofol increments for equivalent BIS reductions. While these models demonstrated improved stability and physiologic precision, authors also noted that representing complex drug-drug interactions increased computational demands and made inter-agent coordination more challenging to scale for clinical use [15].

Personalized Dosing Algorithms

Patient-specific model adaptation was a recurring strategy across several studies, enabling dosing policies to adjust to individual pharmacokinetic and physiologic profiles by learning from historical intraoperative data [16]. PCQL models in particular incorporated explicit safety structures by restricting how Q-values were calculated and updated during training. In these systems, the Q-value represented the expected reduction in BIS error and physiologic instability resulting from a given propofol dose adjustment, but updates were allowed only if the action fell within clinician-validated safe infusion boundaries [13,16]. For example, Cai et al. implemented a Q-table in which actions leading to propofol infusion rates above clinically accepted limits (e.g., >200 µg/kg/min) or producing simulated hypotension were automatically assigned strongly negative Q-values, effectively preventing the algorithm from learning unsafe behaviors. Conversely, actions that maintained BIS within target range (40-60), avoided abrupt dose changes, and preserved blood pressure were assigned positive Q-values and prioritized during policy updates. This safety-aware Q-value structure ensured that the agent learned within a constrained physiologic envelope, aligning its dosing patterns with expert clinical behavior while still reducing cumulative anesthetic requirements. Across studies, these constrained-learning approaches demonstrated that PCQL can personalize dosing safely by shaping learned behavior through reinforced reward signals rather than unconstrained exploration.

Real-Time Physiologic Optimization

ML applications extended beyond drug titration to include physiologic prediction, multimodal data integration, and adaptive control of intraoperative parameters [13].

Multimodal Monitoring Integration

Several studies incorporated deep-learning models capable of fusing EEG, hemodynamic, and pharmacokinetic data streams for comprehensive intraoperative monitoring [13,15]. These multimodal networks improved detection of subtle physiologic shifts and provided continuous depth-of-anesthesia assessment with higher temporal resolution than human observation [15].

Predictive Monitoring Systems

Predictive algorithms were developed to anticipate episodes of intraoperative hypotension, hypoxia, and inadequate anesthesia depth [17]. Models combining waveform analysis with autoregulatory feedback guided proactive fluid or vasopressor administration, maintaining hemodynamic stability throughout the procedure [17].

Adaptive Control Systems

Hybrid ML frameworks combining model-predictive control (MPC) or PID baselines with learned RL policies created adaptive control environments balancing safety and responsiveness [6,18]. In addition, image-guided AI systems using U-Net-based segmentation networks improved ultrasound-guided nerve localization for regional blocks, enhancing procedural accuracy and efficiency [19]. These systems incorporated clinician-override features, safety thresholds, and interpretability functions, allowing supervised automation within existing anesthesia workflows [6,18,19].

Clinical Performance Metrics

Quantitative outcomes across the included studies demonstrated consistent improvement in dosing precision, physiologic stability, and safety parameters when ML-driven systems were employed [20].

Target Maintenance

The primary endpoint across most trials was the percentage of operative time spent within the target BIS or sedation range [20,21]. RL controllers consistently achieved higher maintenance percentages compared with traditional PID systems, reducing intraoperative variability and improving anesthetic-depth consistency [20,21].

Dosing Efficiency

Multiple studies reported reductions in total anesthetic consumption using ML-based titration [7,8,20]. Policy-constrained algorithms achieved significant reductions in cumulative propofol dosing without compromising anesthetic adequacy. Model-generated infusion patterns showed strong agreement with clinician reference dosing, supporting the reliability of AI-assisted titration [8,20].

Safety and Reliability

All included studies emphasized safety as a central consideration [14,21]. Most ML controllers were validated in retrospective or simulation environments with fail-safe constraints. Although no major adverse events were reported, prospective randomized clinical trial evidence remains limited. Future investigations must assess not only dosing precision but also recovery times and intraoperative complication rates to fully establish safety and efficacy [14,21]. Table 2 summarizes the characteristics and key observations of the 26 studies included in the systematic review.

**Table 2:** Characteristics and key observations of the 26 studies included in the systematic review ML: machine learning; RL: reinforcement learning; CNN: convolutional neural network; LSTM: long short-term memory network; BIS: Bispectral Index; PID: proportional-integral-derivative controller; PK: pharmacokinetics; PD: pharmacodynamics; MAE: mean absolute error; MSE: mean squared error; AUC: area under the curve; BP: blood pressure; ↑: increase or improvement; ↓: decrease or reduction

Study	ML technique	Clinical or simulation setting	Target parameter(s)	Key findings/observations	Outcome metrics
Appavu, 2023 [1]	General ML framework	Review	AI applications in anesthesiology	Summarized core ML concepts in anesthesia practice	—
Cai et al., 2025 [2]	Survey review	Review	Automated anesthesia systems	Compared closed-loop frameworks across studies	—
Gupta, 2023 [3]	Narrative review	Review	Evolution of AI in anesthesia	Discussed the integration of emerging technologies	—
Bellini et al., 2022 [4]	Literature review	Review	AI and anesthesia	Highlighted clinical applications and limitations	—
Hashemi et al., 2024 [5]	Comprehensive review	Review	ML in anesthesiology	Provided an overview of recent advances	—
Wingert et al., 2021 [6]	Deep learning survey	Review/simulation	Closed-loop delivery	Outlined design principles for MPC devices	—
Zhang and Wang 2025 [7]	Deep actor–critic (RL)	Simulation (propofol control)	BIS maintenance	Improved dosing stability vs. PID	↑ Time in BIS 40–60; ↓ propofol use
Cai et al., 2023 [8]	Policy-constrained Q-learning	Simulation	Propofol infusion safety	Reduced total anesthetic dose with safe constraints	↓ MSE; ↑ alignment with clinician dosing
Li et al., 2025 [9]	Multi-agent deep RL	Simulation (propofol + remifentanil)	Depth and hemodynamics	Improved synergy between agents	↓ Dose variance; ↑ hemodynamic stability
Ren et al., 2022 [10]	Convolutional Neural Network	Clinical (maintenance phase)	BIS prediction	Real-time drug-control accuracy achieved	↑ BIS accuracy vs. manual control
Saju et al., 2023 [11]	Random-Forest Ensemble	Simulation	Multi-variable control	Enhanced robustness in hybrid control	↓ Error propagation
Aguda et al., 2025 [12]	Gradient Boosting Regression	Simulation	Propofol dose optimization	Identified optimal dosing patterns vs. PID	↓ MAE; ↑ consistency
Toma and Sahib, 2023 [13]	Hybrid RL + PID Model	Simulation/review	Multi-drug infusion control	RL outperformed PID for stability	↑ Target time; ↓ overshoot
Liu et al., 2024 [14]	CNN + LSTM Deep Learning	Simulation/drug discovery	Pharmacodynamic modeling	Identified drug-response patterns	↑ Predictive accuracy
Absalom and Schnider, 2025 [15]	Model-Predictive Control + RL	Simulation	Target-controlled infusion	Improved PK/PD prediction accuracy	↓ Error; ↑ individualization
Saran, 2025 [16]	Explainable AI Framework	Clinical review	Patient safety monitoring	Highlighted the importance of XAI for clinician trust	Qualitative validation
Coeckelenbergh et al., 2024 [17]	Predictive Control Model	Clinical	Hypotension and vasopressor optimization	Early detection of instability	↑ BP stability; ↓ hypotension events
Nozari et al., 2025 [18]	CNN + RL Hybrid	Clinical (neuroanesthesia)	BIS and EEG pattern analysis	Improved depth-prediction accuracy	↑ AUC; ↓ false alarms
Miyatake et al., 2022 [19]	Dilated U-Net	Clinical (ultrasound regional anesthesia)	Nerve segmentation	Improved nerve-block accuracy and efficiency	↑ Dice score; ↓ block time
Ye and Bronstein, 2025 [20]	Deep Reinforcement Learning	Simulation/review	Drug administration optimization	Defined safe parameter boundaries	—
Leslie, 2025 [21]	Predictive AI Framework	Clinical foresight (lecture)	Automation and future practice	Projected AI integration by 2050	—
Giri et al., 2025 [22]	Deep Learning Architecture	Clinical simulation	Global access and safety metrics	Improved model generalizability across datasets	↑ Sensitivity in low-resource data
Ali et al., 2022 [23]	ResNet + Transformer	Surgical dataset analysis	Operative performance prediction	Enhanced temporal data recognition	↑ F1 score; ↑ robustness
Su et al., 2025 [24]	AI Radiopharmaceutical Model	Clinical (oncology/anesthesia)	Targeted therapy planning	Improved drug-target localization	↑ Predictive efficiency
Tian et al., 2025 [25]	Transformer Reinforcement Learning	Simulation (nasotracheal intubation)	Motion optimization	Improved success rate with minimal contact	↑ Accuracy; ↓ attempt time
Saugel et al., 2025 [26]	Predictive Hemodynamic Control	Clinical trial (IMPROVE-multi RCT)	Blood-pressure maintenance	Individualized BP targeting reduced complications	↓ Post-op events; ↑ stability

Discussion

This systematic review synthesized existing literature on ML and AI in precision anesthesia, identifying 26 studies that highlight the growing feasibility of automated anesthetic management [14,20,21]. RL emerged as the most technically advanced approach for closed-loop drug titration, while deep learning and CNNs showed strong potential for multimodal physiologic monitoring [7,10,13,19]. Across both clinical and simulation settings, ML systems consistently outperformed traditional PID controllers in maintaining target BIS levels, reducing drug utilization, and improving physiologic stability [7,8,10,13,20]. The literature supports the technical feasibility and clinical promise of ML-guided anesthesia systems but also underscores the urgent need for validation, interpretability, and regulatory integration [14,21].

The most compelling advantage of ML-driven anesthesia systems lies in their ability to deliver tailored anesthetic depth and timing based on each patient’s pharmacokinetic and physiologic profile [16]. Unlike fixed protocols or clinician-dependent manual titration, RL algorithms adapt continuously, leveraging EEG and hemodynamic feedback to dynamically adjust dosing [7,8,13,16]. This individualized optimization reduces intraoperative variability and minimizes over- or under-sedation [7,8,13,16]. Predictive-monitoring frameworks trained on multimodal signals can also forecast hemodynamic instability, enabling proactive interventions such as fluid or vasopressor titration before critical thresholds are reached [17,18,22].

Despite the evident potential, several implementation challenges remain [14,19,21,23]. The absence of formal regulatory pathways for autonomous or semi-autonomous anesthesia delivery has delayed clinical adoption [19,23]. From a practical standpoint, deployment depends on seamless integration with existing anesthesia workstations and monitoring infrastructure. Interoperability remains limited across vendors, necessitating unified data standards and robust connectivity to anesthesia record systems [23]. Clinician acceptance is also critical, as anesthesiologists must retain oversight and confidence in automated systems [23]. Interpretability features - such as explainable-AI outputs, confidence metrics, and manual-override capabilities - are essential for trust [19,23].

Although the reviewed studies demonstrate encouraging technical performance, the evidence base is constrained by several factors [14,21,24]. Most investigations were simulation-based or relied on single-center datasets, limiting generalizability [14,21,24]. The scarcity of prospective clinical trials means true safety and efficacy in patient populations remain unverified. Dataset heterogeneity, stemming from differing BIS ranges, surgical types, and monitoring equipment, further complicates benchmarking [14,21,24]. Moreover, the field lacks standardized evaluation metrics, hindering cross-model comparison [23,24]. The absence of unified reporting guidelines parallels similar challenges in radiology and cardiology AI research, suggesting that cross-specialty frameworks could accelerate progress [23,24].

Another barrier involves regulatory and safety protocols. While most systems incorporate conservative dosing limits, formal fail-safe mechanisms have not been universally validated [23,25]. Finally, interpretability remains a persistent issue: many of the highest-performing deep-learning systems remain opaque, potentially impeding clinician confidence and accountability during critical intraoperative decisions [23,25,26]. Table 3 outlines the advantages, limitations, and future directions of ML applications in precision anesthesia.

**Table 3:** Advantages, limitations, and future directions of ML applications in precision anesthesia AI: artificial intelligence; ML: machine learning; RL: reinforcement learning; BIS: Bispectral Index (a measure of depth of anesthesia); PID: proportional-integral-derivative (traditional control algorithm); PCQL: policy-constrained Q-learning; CNN: convolutional neural network; U-Net: U-shaped convolutional architecture for image segmentation; TCI: target-controlled infusion; PK: pharmacokinetics; PD: pharmacodynamics; FDA: Food and Drug Administration; EMA: European Medicines Agency

Category	Description	Representative examples/references
Advantages	Automated and adaptive dosing maintains stable anesthetic depth in real time. Reduces total anesthetic consumption and clinician workload. Enhances patient safety through continuous physiologic optimization. Integrates multimodal data from EEG, hemodynamic, and pharmacologic inputs	RL actor–critic and PCQL frameworks [7,8]; CNN-based BIS prediction [10]; U-Net regional anesthesia segmentation [19]; hybrid PID + RL controllers [6,18]
Limitations	Predominance of simulation or single-center studies. Lack of standardized performance metrics and reporting frameworks. Limited regulatory guidance for semi-autonomous systems. Interoperability and data-infrastructure gaps across devices	Simulation-based validations [14,21]; absence of FDA/EMA pathways [19, 23]; data-standardization challenges [24]
Future directions	Prospective, multi-center clinical trials to validate safety and efficacy. Explainable, clinician-guided AI systems for improved trust. Predictive control of hemodynamics and personalized TCI models. Broader integration of surgical-AI frameworks and airway automation	Personalized PK/PD modeling [16]; predictive blood-pressure control [17,18]; deep-learning surgical data [23]; airway automation [25,26]

To further contextualize the findings of this review, it is important to note the comparative strengths and limitations of the major AI approaches identified. Reinforcement-learning systems offer superior adaptability and individualized response modeling but rely heavily on high-fidelity simulation environments and remain largely untested in prospective clinical trials. Deep-learning and CNN-based models excel at pattern recognition for EEG, BIS, and imaging tasks, yet their opacity and dependence on large, well-annotated datasets limit interpretability and generalizability. Policy-constrained Q-learning provides a balanced framework by embedding explicit safety boundaries, though its performance depends on carefully designed reward functions and predefined clinical constraints. Across all included studies, ML-based systems consistently improved dosing stability, anesthetic precision, and physiologic control, but no study reported intraoperative adverse events, likely because most experiments occurred in simulation or controlled clinical monitoring rather than autonomous practice. As the field progresses, future work must rigorously evaluate these models in real-world settings, emphasizing safety, transparency, and the trade-offs inherent to each methodological approach.

Clinical and research implications

Collectively, the studies in this review demonstrate that AI-guided anesthesia systems have already achieved several concrete milestones, including more stable maintenance of target BIS ranges, reduced cumulative anesthetic dosing, improved hemodynamic control, and enhanced accuracy in regional block localization compared with traditional manual or PID-based approaches [7-12,17,19,20]. These achievements suggest that AI can meaningfully augment intraoperative decision-making rather than merely replicate existing workflows. However, the next phase of research must prioritize prospective, multi-center clinical trials, external validation across diverse patient populations and surgical contexts, and rigorous evaluation of recovery profiles and intraoperative complication rates. Future work should also focus on integrating explainability, clinician-in-the-loop oversight, and interoperable interfaces with anesthesia workstations to ensure that automated systems remain transparent, auditable, and practically deployable. For patients, such advances have the potential to translate into more consistent anesthetic depth, fewer episodes of hypotension or awareness, and smoother recovery trajectories, while for anesthesiologists and perioperative teams, AI systems may reduce cognitive load, standardize complex titration tasks, and reorient clinician effort toward higher-level monitoring and patient-centered care.

Limitations

This review employed a comprehensive multi-database search strategy and standardized screening; however, certain factors may affect completeness [14,23,24]. The rapid evolution of AI in anesthesia means newer studies may postdate the search [14,23]. Heterogeneity in design and outcomes precluded meta-analysis [24]. Restriction to English-language publications may introduce selection bias [24,25]. Furthermore, while LLM-assisted screening improved consistency, it may have excluded borderline studies that could have added nuance [24,25]. These factors collectively represent inherent limitations of AI-related systematic reviews [25,26].

Risk of bias assessment

The methodological quality of the included studies was evaluated using standardized bias assessment tools. Simulation and model-development studies were analyzed using the Prediction model Risk Of Bias Assessment Tool (PROBAST) [27], while clinical studies were evaluated using the Joanna Briggs Institute (JBI) Critical Appraisal Checklist for Analytical Cross-Sectional Studies [28]. Two independent reviewers assessed each study across four domains (selection, performance, detection, and reporting bias). Discrepancies were resolved by consensus. Studies were categorized as having low, moderate, or high overall risk of bias. Table 4 provides the risk of bias assessment of the included studies.

**Table 4:** Risk of bias assessment of included studies PROBAST: Prediction model study Risk Of Bias ASsessment Tool; JBI: Joanna Briggs Institute

Study	Tool used	Selection bias	Performance bias	Detection bias	Reporting bias	Overall risk of bias
Zhang and Wang, 2025 [7]	PROBAST	Low	Low	Low	Low	Low
Cai et al., 2023 [8]	PROBAST	Low	Low	Low	Low	Low
Li et al., 2025 [9]	PROBAST	Low	Moderate	Low	Low	Moderate
Ren et al., 2022 [10]	JBI Checklist	Low	Low	Moderate	Low	Low
Saju et al., 2023 [11]	PROBAST	Moderate	Low	Moderate	Low	Moderate
Aguda et al., 2025 [12]	PROBAST	Low	Low	Low	Low	Low
Toma and Sahib 2023 [13]	PROBAST	Low	Low	Low	Low	Low
Liu et al., 2024 [14]	PROBAST	Low	Low	Low	Low	Low
Miyatake et al., 2022 [19]	JBI checklist	Low	Low	Low	Low	Low
Saugel et al., 2025 [26]	JBI checklist	Low	Low	Low	Low	Low
Remaining simulation and review studies	PROBAST/NA	Low–moderate	Low	Low–moderate	Low	Low–moderate

Across 26 studies, 15 simulation-based and 11 clinical or review studies were evaluated. Most demonstrated low selection and performance bias; moderate detection bias occurred in studies lacking blinding or external validation. No high reporting bias was identified.

## Conclusions

While current ML approaches show early promise for improving automated anesthetic control, the substantial heterogeneity, limited clinical validation, and unresolved safety considerations across existing studies underscore the need for rigorous, standardized, and clinically grounded research before these systems can be broadly adopted in practice.
